# Understanding Cortical Dysfunction in Schizophrenia With TMS/EEG

**DOI:** 10.3389/fnins.2020.00554

**Published:** 2020-05-28

**Authors:** Aadith Vittala, Nicholas Murphy, Atul Maheshwari, Vaishnav Krishnan

**Affiliations:** ^1^Department of Biosciences, Rice University, Houston, TX, United States; ^2^Department of Psychiatry and Behavioral Science, Baylor College of Medicine, Houston, TX, United States; ^3^Department of Neurology, Baylor College of Medicine, Houston, TX, United States; ^4^Department of Neuroscience, Baylor College of Medicine, Houston, TX, United States

**Keywords:** TMS/EEG, schizophrenia, cortical correlates, gamma oscillations, cortical inhibition

## Abstract

In schizophrenia and related disorders, a deeper mechanistic understanding of neocortical dysfunction will be essential to developing new diagnostic and therapeutic techniques. To this end, combined transcranial magnetic stimulation and electroencephalography (TMS/EEG) provides a non-invasive tool to simultaneously perturb and measure neurophysiological correlates of cortical function, including oscillatory activity, cortical inhibition, connectivity, and synchronization. In this review, we summarize the findings from a variety of studies that apply TMS/EEG to understand the fundamental features of cortical dysfunction in schizophrenia. These results lend to future applications of TMS/EEG in understanding the pathophysiological mechanisms underlying cognitive deficits in schizophrenia.

## Introduction

Schizophrenia is a debilitating psychiatric disorder with a mean lifetime prevalence of 1% (Kahn et al., 2015). Patients with schizophrenia present with diverse clinical symptoms; core features include positive symptoms such as delusions and hallucinations and negative symptoms such as reduced motivation and social withdrawal. The first episode of psychosis (defined broadly as either hallucinations or delusional behavior) typically occurs in late adolescence or early adulthood (late teenage years or early twenties), frequently preceded by a clinically heterogeneous prodromal phase with varied features such as blunted affect, social withdrawal, delusions, and sub-psychotic perceptual disturbances (Owen et al., 2016). Defining consensus diagnostic features of this prodrome has been the subject of extensive debate (Tsuang et al., 2013). Accurately identifying such at risk individuals may provide insights into the initial neurobiological alterations associated with schizophrenia and also facilitate the testing of specific interventions designed to prevent conversion to frank psychosis (Addington and Heinssen, 2012).

Neuroimaging studies in schizophrenia patients have consistently observed reduced cortical gray matter volume in temporal and prefrontal areas, an anatomical change that may reflect neuronal cell loss, lowered dendritic complexity, and/or synaptic density changes (Karlsgodt et al., 2010; Dietsche et al., 2017). However, such microscopic neuropathological changes are both impractical to measure in a clinical setting and may represent a secondary consequence of underlying network dysfunction. In contrast, neurophysiological measures of cortical function obtained in awake and interacting patients (and/or animal models) may offer a more practical biomarker of specific cognitive deficits while also highlighting themes of cellular and/or molecular dysfunction (Linden, 2012). One example (which we discuss further below) is gamma rhythms: parvalbumin-expressing, fast-spiking inhibitory interneurons (PV-INs) generate gamma frequency oscillations through phase-locked inhibitory firing, which may aid in the coordination of local information processing and is modulated in a top-down fashion (Uhlhaas and Singer, 2013). Altered gamma rhythms have been implicated as a substrate for several cognitive deficits of schizophrenia and remain popular as a major clinical biomarker (Gonzalez-Burgos et al., 2015).

Transcranial magnetic stimulation (TMS) has emerged as a powerful tool to non-invasively study cortical physiology in humans (Hallett, 2000; Valero-Cabré et al., 2017). While early applications of this technology were restricted to measuring or mapping motor cortex activity, the development of compatible electroencephalography (EEG) techniques has led to the growth of combined TMS/EEG to measure and manipulate motor and non-motor cortical function (Daskalakis et al., 2012; Tremblay et al., 2019). In contrast to other non-invasive modalities such as functional magnetic resonance imaging (fMRI), the superior temporal resolution of TMS/EEG offers a unique window into deficits in cellular function and synaptic transmission across cortical networks (Premoli et al., 2014; Kaskie and Ferrarelli, 2018; Hui et al., 2019). In this review, we will introduce the lay reader to general concepts pertaining to TMS/EEG and review recent studies that have employed TMS-EEG to directly measure cortical dysfunction in patients with schizophrenia.

## Applying TMS/EEG

Transcranial magnetic stimulation uses an electromagnetic coil to create a time-varying magnetic field within the cortex. This changing magnetic field then induces an electrical current, transiently affecting the firing of cortical neurons close to the coil (Tremblay et al., 2019). Thus, TMS produces a spatiotemporally localized change in neural activity. Through assessments of behavioral measures (e.g., muscle twitching) and local neural recordings, the extent of direct neural modulation has been found to be approximately 2 mm in radius (Romero et al., 2019). TMS-induced magnetic fields do not penetrate subcortical structures, limiting the direct and selective stimulation of deep brain regions. Nevertheless, these regions may be modulated indirectly by stimulating a functionally connected cortical region (McClintock et al., 2011).

**FIGURE 1:**
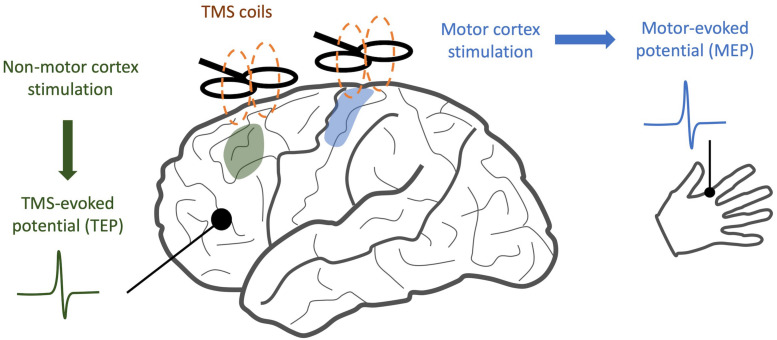
Comparison of TMS/EEG and traditional TMS. While traditional TMS techniques have focused on areas with readily observable responses, such as stimulation of motor cortex and detection of motor-evoked potentials, TMS/EEG allows us to observe cortical responses to stimulation with much greater spatial resolution.

When TMS is applied to the motor cortex, electromyography (EMG) of the associated muscle shows a motor-evoked potential (MEP) (Bestmann and Krakauer, 2015). If TMS is applied to a non-motor area, there may be no MEP, but a TMS-evoked potential (TEP) can be visualized through simultaneously recorded EEG. This TEP is a surface representation of cortical activity in response to the magnetic pulse (Figure 1). After incorporating special methodological considerations to account for the auditory and somatosensory components of TMS stimuli (Conde et al., 2019), TEPs provide a quantitative measure of cortical information spread across multiple domains. When applied to patients with neuropsychiatric illness, changes in the time course and frequency spectrum of TEP responses may indicate disease- or treatment-related alterations in neural oscillations and cortical inhibition. Similarly, when compared along the spatial domain, altered TEPs provide insights into changes in connectivity between brain regions (Rogasch et al., 2014).

## Altered Neural Oscillations

Neural oscillations are rhythmic fluctuations in the electrical activity of populations of neurons, and these synchronized oscillations may allow more efficient communication between different brain regions (Uhlhaas et al., 2009; Buzsáki and Schomburg, 2015). Neural oscillations can be measured via extracranial EEG and resolved into specific bands of frequency: delta (1–3 Hz), theta (4–7 Hz), alpha (8–12 Hz), beta (13–30 Hz), and gamma (31–80 Hz) (Mathalon and Sohal, 2015). These synchronized oscillations are correlated with frequency-specific cognitive functions in healthy individuals (Uhlhaas, 2009), suggesting a possible link between the processes underpinning dysfunctional neural oscillations and the cognitive deficits seen in schizophrenia (Uhlhaas and Singer, 2013). Many EEG-based studies have identified disruptions in gamma band oscillatory power in schizophrenia patients (see Hunt et al., 2017 for a review of these findings). Unfortunately, these findings have varied in directionality, suggesting that baseline alterations in gamma band power may not be themselves predictive of schizophrenia. In contrast, the finding of reduced task-based gamma power in schizophrenia patients has been more consistent (Hunt et al., 2017; Nguyen et al., 2020). Tasks such as the auditory steady state response (ASSR) may provide a robust measurement of gamma oscillations in the auditory cortex (O’Donnell et al., 2013), whereas the Stroop and N-Back tasks can be used to measure synchrony across multiple bands (Barr et al., 2017; Popov et al., 2019).

Relative to the study of strictly task-based biomarkers, TMS/EEG offers a more direct and spatially localized probe of cortical oscillations. In one early TMS/EEG study, schizophrenia patients and healthy controls underwent single-pulse TMS at the premotor cortex, and high-density EEG showed that evoked gamma oscillations in frontal areas of schizophrenia patients were significantly decreased in amplitude and synchronization (Ferrarelli et al., 2008). This finding has been replicated in an independent TMS/EEG study (Canali et al., 2015). However, patients in both studies were receiving chronic antipsychotic therapy. A more recent TMS/EEG study on medication naive first-episode psychosis patients found similar reductions in the amplitude of evoked low gamma (27–33 Hz) oscillations (Ferrarelli et al., 2019).

Similar findings have been observed in preclinical models of schizophrenia; in two separate putative mouse models of schizophrenia (chronic ketamine administration and 22q11.2-analog deletion), mice displayed a deficit in evoked gamma band activity together with deficits in the reliability of neuronal coactivity patterns that could not be explained by alterations in single neuron activity. Interestingly, acute ketamine administration or acute pharmacogenetic suppression of PV-INs did not have the same effect (Hamm et al., 2017), suggesting the presence of long-standing network adaptations involving PV-INs.

Resonant frequencies offer a complimentary approach to studying neural oscillations in schizophrenia. Upon a pulse of TMS stimulation, different cortical areas tend to oscillate at specific frequencies, with more frontal areas oscillating at higher frequencies and more posterior regions oscillating at lower frequencies. Even as the intensity or location of the TMS pulse is varied, the EEG signal at each region continues to oscillate at the same frequency. Thus, region-specific resonant frequencies appear to be a deliberate physiological property reflecting variations in regional neuronal organization and thus may offer a useful tool for studying cortical region-specific disturbances (Rosanova et al., 2009). In one study, resonant frequencies in frontal regions were lower in schizophrenia compared with healthy controls, with the largest deficits seen in the prefrontal cortex, and these deficits correlated with positive symptom severity (Ferrarelli et al., 2012). Another study applied the same technique to patients with schizophrenia, major depression, and bipolar disorder, and found that reductions in resonant frequencies in frontal regions were shared among all three disorders (Canali et al., 2015). Thus, alterations in resonant frequencies as identified by TMS/EEG may correlate with core cognitive deficits across a number of different psychiatric disorders (Etkin et al., 2013), but larger multi-center studies need to be conducted to confirm these findings. Taken together, these studies support the potential of TMS/EEG in better understanding dysfunctional oscillations in schizophrenia. Further research is required to discern how these measures may assist in differentiating between schizophrenia and other psychiatric disorders.

## Altered Neural Inhibition

Gamma oscillations are produced by the firing patterns of inhibitory PV-INs, cells which play a role in maintaining an appropriate excitatory/inhibitory balance in the cortex in mice (Sohal et al., 2009; Ferguson and Gao, 2018). Thus, schizophrenia-associated alterations in either baseline or evoked gamma activity in frontal cortical regions may be related to deficits in frontal PV-IN function (Uhlhaas and Singer, 2013). In support of this hypothesis, a meta-analysis of post mortem studies has found an approximately 30% reduction in PV-IN density within pre-frontal regions (Kaar et al., 2019). In line with these findings, TMS/EMG studies that examine motor cortex inhibition in humans have indeed shown a consistent disinhibition effect in schizophrenia (Bunse et al., 2014), and this effect has been replicated by research on non-motor areas using TMS/EEG (Kaskie and Ferrarelli, 2018).

To study cortical inhibition via TMS/EEG, many studies make use of a paired-pulse paradigm. Two separate TMS pulses (the conditioning stimulus and the test stimulus) are applied to the cortex; if they are applied within a 1–4 ms of each other, the response to the test pulse is typically diminished relative to the conditioning pulse and termed short interval intracortical inhibition (SICI). In contrast, with intervals set to 50–200 ms, the resulting inhibition is called long interval intracortical inhibition (LICI) (Rogasch et al., 2014). These two inhibitory responses are thought to require two distinct gamma-aminobutyric acid (GABA) receptors: GABAA mediates SICI and GABAB mediates LICI, and they can be differentiated by receptor-specific ligands (McClintock et al., 2011; Premoli et al., 2014).

Recent studies have applied paired-pulse TMS/EEG to measure inhibition of TEPs in the prefrontal cortex (Cash et al., 2017). One study investigated LICI in the dorsolateral prefrontal cortex of schizophrenia patients, OCD patients, and healthy controls (Radhu et al., 2015). They found a deficit in prefrontal cortex LICI specific to schizophrenia patients, suggesting that it might represent a biomarker for GABAB receptor dysfunction in schizophrenia. Interestingly, the study was not able to find any differences in motor cortex LICI between the three groups, raising the possibility that schizophrenia may impart varying detrimental effects on regional circuitry (Radhu et al., 2015). A follow-up study characterized prefrontal LICI in first-degree relatives of schizophrenia patients; in this at-risk group, measures of LICI appeared to be intermediate between the levels for healthy controls and schizophrenia patients. However, this difference was not statistically significant, so further study is warranted (Radhu et al., 2017). Prefrontal SICI may also be deficient in schizophrenia. One study applied paired-pulse TMS/EEG to the dorsolateral prefrontal cortex of schizophrenia patients and healthy controls, and the results showed that SICI was reduced in schizophrenia. This deficit was also correlated with a measure of working memory (Noda et al., 2017). Two other studies employing motor cortex TMS identified similar findings (Takahashi et al., 2013; Bridgman et al., 2016). While encouraging, all of these studies used relatively small sample sizes and did not control for chronic antipsychotic use. Future studies need to extend these results to larger populations (including unmedicated patients) to better understand schizophrenia-related changes in intracortical inhibition at a circuit level. In addition, overall changes in SICI and LICI may not be specific to schizophrenia (Radhu et al., 2013; Jeng et al., 2019), but region-specific changes in these biomarkers of cortical inhibition may help further distinguish these various disorders (Menzies et al., 2007; Hashimoto et al., 2008; Thompson et al., 2009; Luscher et al., 2011; de Jonge et al., 2017).

## Altered Connectivity

In addition to alterations in excitation/inhibition imbalance, schizophrenia has also been broadly conceptualized as a failure of efficient communication between neural systems required for cognition and perception. Resting state fMRI analyses have provided strong support along these lines, demonstrating alterations in functional connectivity between the prefrontal cortex and other brain regions (including the temporal lobes, hippocampus, and striatum) in patients with schizophrenia (Schmitt et al., 2011; Zhou et al., 2015; Dong et al., 2018). In addition, EEG studies (without TMS) have found impairments in synchrony between gamma band neural oscillations across various brain regions (Wynn et al., 2015; Schulz et al., 2017; Steinmann et al., 2017; Brennan et al., 2018). Similar deficits have been observed in genetic mouse models of schizophrenia; one study found decreased prefrontal-hippocampal theta band synchronization in mice during a working memory task (Sigurdsson et al., 2010). Since neural synchrony is essential for effective communication between neural circuits, synchronization deficits and functional connectivity alterations are likely co-dependent (Uhlhaas and Singer, 2010). With TMS/EEG applied to human subjects, it is possible to study how alterations in synchrony and functional connectivity between cortical regions correlate with specific symptoms of schizophrenia.

To measure the functional connectivity between two cortical regions with TMS/EEG, studies can apply a TMS pulse to one area and then use the EEG data to determine how that pulse spread to another region. Unlike approaches that rely on temporally correlated patterns of activity (e.g., EEG coherence analyses or resting state fMRI), responses to experimentally induced TMS pulses can provide inferences about causality and directionality of cortical information spread (Hallett et al., 2017). One study delivered TMS pulses to the motor cortex and identified more prolonged and recurrent evoked excitation waves in schizophrenia patients compared to healthy controls. This excess cortical activation was also correlated with positive symptoms of schizophrenia (Frantseva et al., 2014). Another TMS/EEG study found similar results when they applied subthreshold pulses to the left motor cortex and found evidence of excess connectivity both within the motor cortex and between the motor cortex and other brain regions in schizophrenia patients (Gupta et al., 2019). Connectivity deficiencies may also be present in some regions of the brain in schizophrenia: a third study applied TMS pulses separately to prefrontal, premotor, motor, and parietal cortex and used EEG source modeling to measure significant current scattering (a proxy for intracortical connectivity). In this study, schizophrenia was associated with lower significant current scattering in prefrontal and premotor areas, and this parameter alone provided good sensitivity and specificity in separating schizophrenia patients from controls (Ferrarelli et al., 2015). A final study identified reduced interhemispheric facilitation between the premotor cortex and the contralateral motor cortex in schizophrenia patients, and they noted that deficits in facilitation were positively correlated with negative symptom burden (Ribolsi et al., 2011). All four of these studies provide evidence for dysfunctional connectivity in schizophrenia, whether interhemispheric or intracortical, as a unifying feature of schizophrenia. However, some of these analyses again included patients on chronic antipsychotic medication, which may lead to EEG alterations, so future studies should ensure inclusion of first-episode psychosis patients to remedy this limitation.

By transiently modifying oscillatory activity, TMS/EEG can offer causal insights into the role of beta/gamma desynchronization in schizophrenia. For example, one study applied TMS at varying frequencies to the prefrontal cortex and simultaneously measured memory formation in healthy subjects. Memory formation was most impaired when the cortex was stimulated at a beta band frequency, implicating desynchronization of beta activity in the process of normal memory formation (Hanslmayr et al., 2014). Similar experiments in schizophrenic patients could help characterize the role of gamma activity in cognitive deficits or hallucinations observed in schizophrenia. Numerous EEG-only studies have suggested these correlations (Andreou et al., 2015; Steinmann et al., 2017; Takahashi et al., 2018), but causality can be better assessed with TMS/EEG.

## Discussion

**FIGURE 2:**
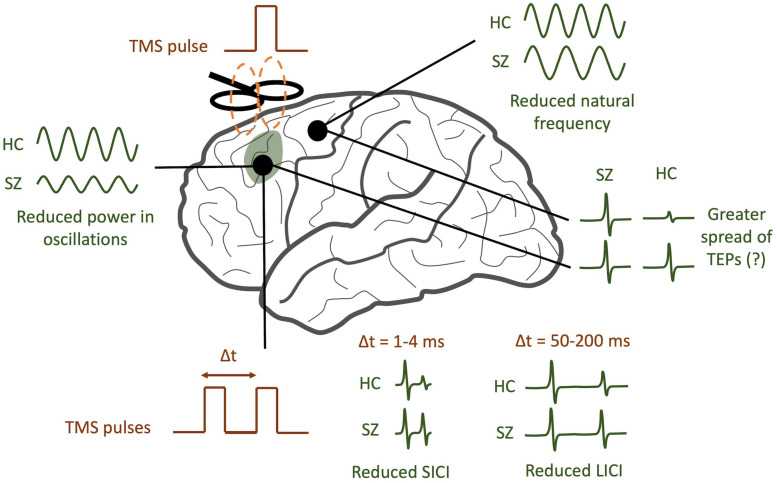
TMS/EEG correlates of schizophrenia (SZ). SZ patients tend to have reduced power in evoked gamma band oscillations compared to healthy controls (HC). In addition, the natural frequency of frontal regions tends to be reduced. A greater spread of TMS-evoked potentials (TEPs) may be observed in SZ, though this finding is still controversial. Finally, SZ patients tend to have reduced short-interval intracortical inhibition (SICI) and reduced long-interval intracortical inhibition (LICI).

In the ways described above, TMS-EEG offers a unique opportunity to directly test a variety of specific hypotheses pertaining to cortical dysfunction in schizophrenia. By analyzing TEPs across multiple domains (see Figure 2 for a graphical abstract of these findings), TMS/EEG may serve to complement insights obtained from other techniques like fMRI and EEG. Several questions remain to be answered. Do the modified resonant frequencies seen in schizophrenia play a causal role in producing specific symptoms, or are they an artifact of treatment? Is there a causal relationship between neural synchronization deficits and the cognitive symptoms of schizophrenia, and what direction does this relationship have? What aspects of TMS/EEG abnormalities in schizophrenia are most predictive and specific to the disorder, and which can be used as diagnostic or prognostic biomarkers? To address these questions, larger multi-center TMS/EEG studies with medication-naïve patients are required in order to both validate findings and provide more statistical strength for hypothesis testing. TMS/EEG studies on patients with other major psychiatric disorders, like depression, bipolar disorder, and anxiety, will help identify signatures that are unique to each of these disorders. Future studies that combine fMRI with TMS/EEG may shed light on how task-related changes in cortical activity may be richly modulated by a variety of subcortical regions (Jung et al., 2020). Finally, as a neurophysiological modality that can be scaled down to mice (Zhang et al., 2019), TMS/EEG may also provide a platform for translationally sound preclinical assessments of treatment and genetic risk.

## Author Contributions

All authors discussed the reviewed research, contributed to manuscript revision, and approved the submitted version. AV wrote the first draft of the manuscript.

## Conflict of Interest

The authors declare that the research was conducted in the absence of any commercial or financial relationships that could be construed as a potential conflict of interest.
